# Drug-induced reactive oxygen species (ROS) rely on cell membrane properties to exert anticancer effects

**DOI:** 10.1038/srep27439

**Published:** 2016-06-09

**Authors:** Hamid R. Molavian, Aaron Goldman, Colin J. Phipps, Mohammad Kohandel, Bradly G. Wouters, Shiladitya Sengupta, Sivabal Sivaloganathan

**Affiliations:** 1 Department of Applied Mathematics, University of Waterloo, Waterloo, Ontario, N2L 3G1, Canada; 2 Department of Medicine, Harvard Medical School, Boston, MA 02115, USA; 3 Harvard-MIT Division of Health Sciences and Technology, Cambridge, MA 02139, USA; 4 Division of Engineering in Medicine, Department of Medicine, Brigham and Women’s Hospital, Boston, MA 02115, USA; 5 Ontario Cancer Institute and Campbell Family Institute for Cancer Research, Princess Margaret Cancer Centre, University Health Network, Toronto, ON M5T 2M9, Canada; 6 Department of Radiation Oncology, University of Toronto, Toronto, ON, M5S 3E2, Canada; 7 Center for Mathematical Medicine, Fields Institute for Research in Mathematical Sciences, Toronto, Ontario M5T 3J1, Canada

## Abstract

Pharmacological concentrations of small molecule natural products, such as ascorbic acid, have exhibited distinct cell killing outcomes between cancer and normal cells whereby cancer cells undergo apoptosis or necrosis while normal cells are not adversely affected. Here, we develop a mathematical model for ascorbic acid that can be utilized as a tool to understand the dynamics of reactive oxygen species (ROS) induced cell death. We determine that not only do endogenous antioxidants such as catalase contribute to ROS-induced cell death, but also cell membrane properties play a critical role in the efficacy of ROS as a cytotoxic mechanism against cancer cells vs. normal cells. Using *in vitro* assays with breast cancer cells, we have confirmed that cell membrane properties are essential for ROS, in the form of hydrogen peroxide (H_2_O_2_), to induce cell death. Interestingly, we did not observe any correlation between intracellular H_2_O_2_ and cell survival, suggesting that cell death by H_2_O_2_ is triggered by interaction with the cell membrane and not necessarily due to intracellular levels of H_2_O_2_. These findings provide a putative mechanistic explanation for the efficacy and selectivity of therapies such as ascorbic acid that rely on ROS-induced cell death for their anti-tumor properties.

Natural products have been extensively investigated for their antitumor properties^1^,^2^. One example is ascorbic acid, better known as vitamin C, which displays selective cancer cell killing behavior that leaves normal cells intact^3^,^4^. Preclinical and clinical trials have repeatedly demonstrated the therapeutic effect of ascorbic acid on various cancer types without any reported side effects to normal tissue^5^,^6^,^7^,^8^,^9^,^10^. One potential explanation for the therapeutic efficacy of ascorbic acid is the elevated concentration of highly membrane-permeable reactive oxygen species (ROS) in the form of H_2_O_2_ on the order of 100 *μ*M in the extracellular space^3^,^11^. In fact, measurements of the concentration of ascorbic acid and H_2_O_2_, both in culture and *in vivo*, suggest that a linear relationship exists between these two concentrations^3^,^11^. These results suggest that ascorbic acid may be a promising candidate for cancer therapy. However, the relationship between ascorbic acid-induced ROS production and cell death remains underexplored.

Cellular antioxidants provide the defensive mechanism against the toxicity of natural products by detoxifying hydrogen peroxide^12^,^13^,^14^,^15^. Indeed, activity of the enzyme catalase that breaks down H_2_O_2_ into its inert components plays a key role in determining the cancer killing ability of natural products such as ascorbic acid^15^. For example, Klingelhoeffer *et al*. observed that when H_2_O_2_ is applied to different cell types, cell viability decreases as the concentration of catalase decreases^16^. Other evidence suggests that antioxidants in combination with ascorbic acid diminish the therapeutic potential of ascorbic acid^17^. These studies provide mounting evidence that ROS and antioxidants are key determinants of the cancer cell killing efficacy of natural products such as ascorbic acid. However, some other studies suggest that there is a lack of correlation between cell viability and concentration levels of other antioxidants such as glutathione and glutathione peroxides^3^,^16^. Hence, the exact mechanism behind the contribution of H_2_O_2_ to cell death remains to be determined.

In this study, we investigated how ROS contributes to cell death and used ascorbic acid as the basis for our model. Utilizing kinetics based on known scientific literature of ascorbic acid activity in cancer cells and normal cells, we developed a mathematical model that predicted a mechanism of cell death via induction of H_2_O_2_. We determined that cell membrane properties play a critical role in H_2_O_2_-driven cell death arising after treatment with ascorbic acid. We then tested these predictions by modulating the permeability of cell membranes with cholesterol or mild detergents and tested intracellular ROS or cell death following exposure to exogenous H_2_O_2_. Surprisingly, we identified that decreased membrane permeability leads to increased cell death, suggesting that H_2_O_2_ exerts anticancer effects through extracellular rather than intracellular mechanisms, presumably by protein and lipid peroxidation. These findings shed light on the mechanisms of drugs that rely on the production of ROS to potentiate anticancer effects.

## Materials and Methods

### Chemicals, reagents and cell lines

Unless otherwise noted, chemicals and reagents were of the highest purity and purchased from Sigma Aldrich (St Louis MO). MDA-MB-231 breast cancer cells were purchased from ATCC and tested for contaminating bacteria prior to performing experiments and cultured in DMEM containing 10% fetal bovine serum (FBS, Life Technologies, Grand Island NY). Prior to experimentation, cholesterol was dissolved directly into cell culture media at working concentration (100 μM) and then sterile filtered. For certain studies, FITC was conjugated to cholesterol by NHS cross linking to cholesterol using standard procedure (Life technologies, Grand Island NY).

### Mathematical Modeling

To understand the cancer cell killing behavior of ascorbic acid, we start by considering the chemical reactions that govern the cell’s defensive mechanisms against H_2_O_2_. Cells employ their detoxifying machinery to protect themselves against H_2_O_2_ damage, primarily in the form of two antioxidant systems, catalase and glutathione peroxidase. These mechanisms remove H_2_O_2_ via the following chemical reactions,


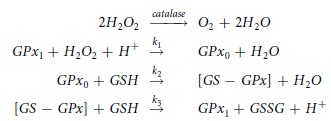


where *k*_1_, *k*_2_ and *k*_3_ are the reaction rates of the glutathione peroxidase system with values of 2.1 × 10^7^ × 10^7^ *M*^−1^ *s*^−1^, 4 × 10^4^ *M*^−1^ *s*^−1^ and 1 × 10^7^ *M*^−1^ *s*^−1^ respectively^18^, (Table 1S, Supplemental Infromation). The molecules in the reactions above, GPX1, GPX0, [GS-GPX] and GSSG, denote reduced glutathione peroxidase, oxidized glutathione peroxidase, glutathione-enzyme complex and glutathione disulfide respectively. Here we are interested in investigating the interplay between the extracellular H_2_O_2_ concentration and the relevant antioxidant concentrations. Although the strength of peroxiredoxins in detoxifying H_2_O_2_ could be as important as the GPx family, we have not included them in these calculations to avoid unnecessary complexity. In the Results and Discussion section we offer an explanation for why, overall, this simplification should not affect our results. We write down the kinetic equations for the concentrations of GSH, GPX1, GPX0, [GS-GPX], and H_2_O_2_ and subsequently obtain the steady state of these equations in the presence of a constant production term for H_2_O_2_ (see below).

Here we denote the intracellular concentrations of GSH, GPX1, GPX0, [GS-GPX], H_2_O_2_ and catalase by *C*_*gsh*_, *C*_*gpx*1_, *C*_*gpx*0_, *C*_*gsgpx*_, *C*_*h*2*o*2_ and *C*_*cat*_ respectively, and the total intracellular production rate of H_2_O_2_ by *P*_*h*2*o*2_. We also assume that during the course of the reactions the sum of the concentrations of GPX1, GPX0, [GS-GPX] remains constant and we denote this by 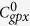. The kinetic equations based on the aforementioned chemical reactions for detoxifying H_2_O_2_ by catalase and GSH are given in Supplemental Information.

Based on experimental results, we assume that the application of ascorbic acid results in an increase in the extracellular concentration of H_2_O_2_^11^, denoted by 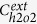. If the intracellular concentration of H_2_O_2_ is 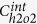 then, assuming a standard diffusive membrane transport term, the rate of H_2_O_2_ transfer into the cell due to ascorbic acid is given by


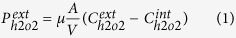


where *μ* is the cell permeability to H_2_O_2_, *A* is the cell surface area and *V* is the cell volume.

The total production rate of H_2_O_2_ inside the cell, *P*_*h*2*o*2_, can be written as follows,


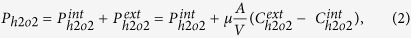


where 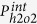 is the contribution of intracellular sources to H_2_O_2_ production and 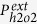 is the rate of transport across the cell membrane given by Eq. (1).

We solve the kinetic equations in steady state with the production rate of Eq. (2) and solve the equations analytically to derive the concentration of H_2_O_2_ in terms of the concentration of other species. For high production rates of H_2_O_2_, which is the case when cells are in a high concentration of ascorbic acid, we obtain the following equation (in this equation we separate the effects of external and internal sources of H_2_O_2_)


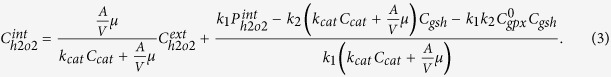


Assuming that the extracellular concentration of H_2_O_2_ depends linearly on the concentration of ascorbic acid^2^,^11^ we obtain the following equation,


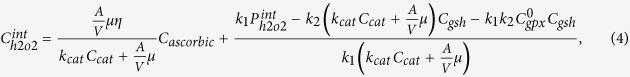


where *C*_*ascorbic*_ is the concentration of ascorbic acid and *η* is the proportionality constant between the concentration of H_2_O_2_ and ascorbic acid, i.e. 
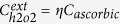
.

In the above model, we assumed that catalase and glutathione peroxidase are the primary antioxidants in the cells and the production rate of H_2_O_2_ is constant. Furthermore, we assume that cell death can depend on the intercellular concentration of H_2_O_2_ and also on the concentration of H_2_O_2_ in the cell membrane. Here we assume, as an illustrative case, that this relation is linear,


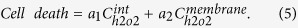


In addition, we assume that 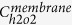 is proportional to 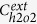. However, we should mention that perturbations in this functional relationship between cell death and intercellular and membrane H_2_O_2_ concentrations do not qualitatively change the results obtained.

### Detection of intracellular reactive oxygen species (ROS)

Prior to treatments, cells were washed and incubated with 2 μM CM-DCFDA (Life technologies, Grand Island NY) for 10 minutes followed by a wash in PBS and then recovery in DMEM for 15 min. Cells were then treated as described in figure legend. After trypsinizing, single cells were processed by flow cytometry to detect DCFDA fluorescence and mean fluorescence intensity was calculated as % increase of vehicle control.

### Cell viability assays

Following treatments described in figure legends, cells were washed 1 time and recovered in serum-free and phenol red-free DMEM and incubated with MTS ONE solution (Promega, Madison WI) following manufacturer protocol.

### Microscopy

Cells were plated in 4 chamber glass slides (BD Biosciences, San Jose CA) at a concentration of 100,000 cells/ml and treated as indicated. Fluorescent images were obtained using three channels (DAPI, FITC and TRITC) on a NIKON Eclipse TI-U microscope with a 20× ELDW objective lens (Nikon, Melville NY). NIS Elements Viewer version 3.22 (Nikon, Melville NY) software was used to capture the images to file.

## Results and Discussion

We sought to investigate the roles of key parameters, as suggested by our model, on the therapeutic effect of ascorbic acid. Based on the parameters included in our model, contained in Eqs (3, 4, 5), different cancer cell killing scenarios can arise, depending on (a) internal production rates of H_2_O_2_ (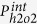) that affect the intrinsic concentration of H_2_O_2_, (b) concentrations of catalase, glutathione peroxidase and GSH, (c) cell membrane properties that vary for different types of cancer cells and normal cells, or some combination of these.

**Figure 1:**
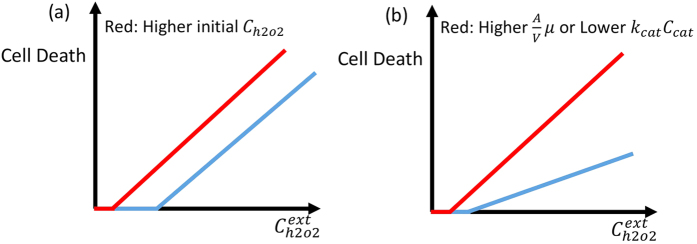
Schematic view of cell death as a function of extracellular concentration of H_2_O_2_. (**a**) In this plot cell permeability and catalase activity are the same for blue and red curves, but the intrinsic concentration of intracellular H_2_O_2_ is higher for the blue curve. (**b**) In this plot cell permeability is lower or catalase activity is higher for blue curve as compared to the red curve and the initial concentration of H_2_O_2_ is a bit more for the blue curve. The difference in catalase activity or cell permeability changes the slope of cell death as a function of the external concentration of H_2_O_2_.

Based on Eqs 3, 4, 5, cell death has to increase linearly as a function of the external concentration of H_2_O_2_ or ascorbic acid if the intracellular concentration of H_2_O_2_ is responsible for the cell death. The proportionality constant depends on the concentration of catalase and the permeability of the cell to H_2_O_2_ (see Eqs (3) and (4)). The internal production of H_2_O_2_ does not have any effect on the proportionality constant and merely shifts the initial cell death at low external concentration of H_2_O_2_. In Fig. 1a,b we plot the effect of higher internal production rate of H_2_O_2_ and, different concentrations of catalase or cell permeability on cell death, respectively. We use these figures as a reference to compare with the experimental results.

**Figure 2:**
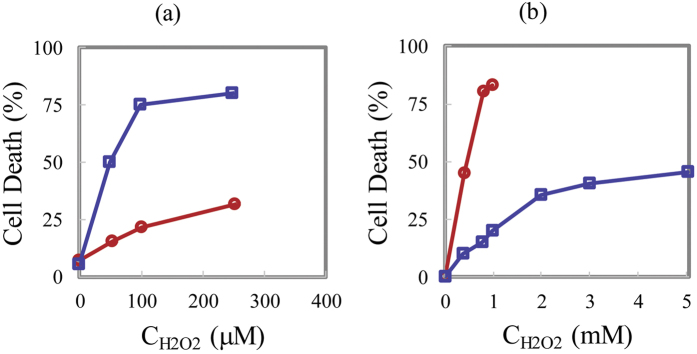
(**a**) Cell death as a function of the concentration of external H_2_O_2_ in human Burkitt’s lymphoma cells (blue squares), normal lymphocytes (red circles) pro-drug^3^. (**b**) Cell death for S. cerevisiae cells as a function of external H_2_O_2_ in stationary phase wild-type (blue squares), exponential phase wild-type cells (red circles)^19^. The stationary and exponential phases exhibit different cell permeabilities with the cell permeability in the exponential phase being higher^19^.

In Fig. 2a we plot the experimental data of cell death for cancer and normal cells as a function of (external) H_2_O_2_ concentration (the data is extracted from)^3^. This data shows linear behavior, which is consistent with our model, which saturates for cancer cells at a maximal cell death of 75%. Interestingly the slope of the lines for cancer and normal cells are clearly different, which based on our qualitative results (Fig. 1) is due to different concentrations of catalase or due to differences in cell membrane properties such as cell permeability or lipid concentration (See also Fig. 1S, Supplementary Information). In this ref. 3, no correlation was observed between cell death and catalase activity, glutathione concentration or glutathione peroxidase activity. Therefore, this again suggests that another parameter is involved and in all likelihood is responsible for the change in cell death. Based on our results, the additional factor that should be taken into account is cell membrane permeability.

To provide additional evidence that cell membrane permeability could be one of the key players in ascorbic acid-induced cell death, we present the results from ref. 19 in Fig. 2b. In this figure cell death is plotted as a function of the concentration of H_2_O_2_ for the stationary and exponentially growing S. cerevisiae cells. Based on the presented results in this paper, the difference between the stationary and growing cells is due to the apparent difference in permeability to H_2_O_2_ between stationary and exponential phase cells. Clearly, Fig. 2a,b show a similar pattern, which provides qualitative evidence that cell membrane properties such as permeability may be a primary factor underlying the experimentally observed differences in cancer cell behavior under conditions of high concentration levels of external H_2_O_2_ (or ascorbic acid).

**Figure 3:**
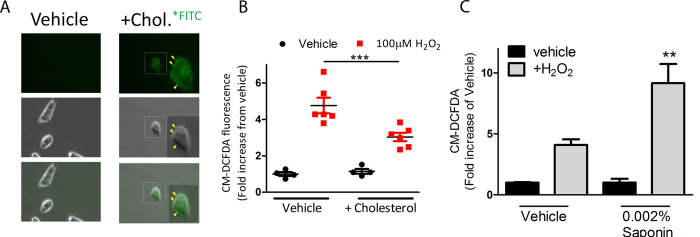
Altering the membrane permeability of breast cancer cells using light detergent or cholesterol affects uptake of ROS. (**A**) Representative bright field and fluorescent microscopy shows MDA-MB-231 cells treated with cholesterol tagged with fluorescein or vehicle control for 2 hours before washing off excess. Fluorescent microscopy indicates localization of cholesterol in the membrane of cells (yellow arrows). (**B**) Quantification of flow cytometric data shows % increase of intracellular ROS following incubation with cholesterol (2 h) and 100 μM H_2_O_2_ (30 min) as determined by activity of the fluorescent probe, CM-DCFDA. (**C**) Quantification of flow cytometric data shows % increase of intracellular ROS following incubation with 0.002% saponin (2 h) and 100 μM H_2_O_2_ (30 min) as determined by activity of the fluorescent probe, CM-DCFDA.

Next, we sought to explore if membrane permeability (first term in Eq. 5) or other properties of the cell membrane (second term in Eq. 5) affects H_2_O_2_-induced cell death. To test this, we first wanted to identify a method of modulating membrane permeability. For decades it has been known that intramembrane cholesterol leads to decreased membrane fluidity and therefore decreased membrane permeability^20^. In fact, increased cholesterol and decreased membrane fluidity is associated with progression of normal breast cells to neoplastic growth^21^. Using fluorescent microcopy, we confirmed that addition of FITC-conjugated cholesterol directly into cell culture will embed into the membrane surface of MDA-MB-231 breast cancer cells (Fig. 3A, yellow arrows). We next wanted to test the effect of increased membrane cholesterol on permeation of H_2_O_2_. Therefore, we treated 231 cells with the cytosolic-localized fluorescent probe, CM-DCFDA, which detects changes in ROS by increasing fluorescence. As expected, we identified that addition of H_2_O_2_ led to an increase of overall ROS, indicated by increased DCFDA fluorescence as determined by flow cytometry (Fig. 3B). However, by studding cholesterol into the membrane of cells, the overall increase of intracellular ROS was significantly diminished compared to vehicle control (p < 0.001). These results confirmed that decreased membrane permeability leads to decreased H_2_O_2_ uptake from extracellular source. In a separate experiment, we modified membrane permeability using a light detergent (0.002% saponin), which confirmed that increasing membrane permeation enhanced uptake of H_2_O_2_ (Fig. 3C).

**Figure 4:**
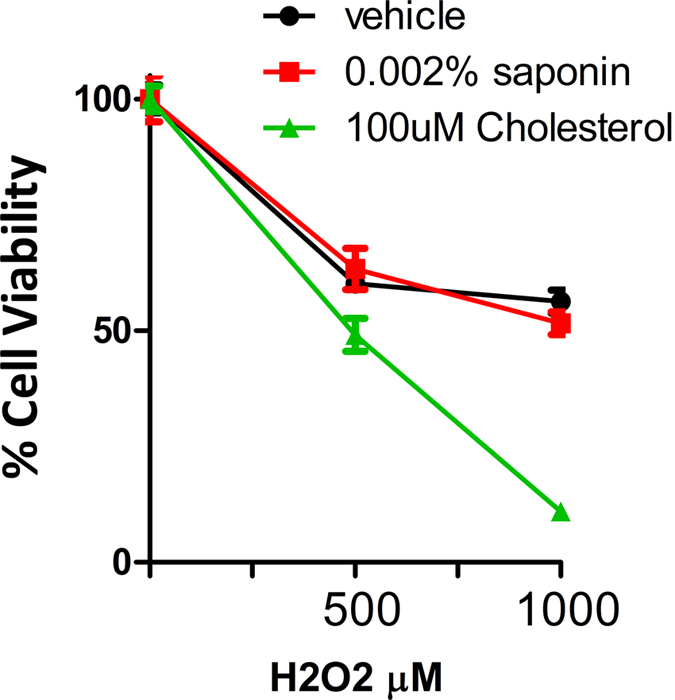
Decreased but not increased cell membrane permeability leads to enhanced cell killing by hydrogen peroxide. Cell viability analysis of MDA-MB-231 shows the cytotoxic effect of hydrogen peroxide on cells with increased permeability (+saponin) or decreased permeability (+cholesterol).

Finally, we tested how membrane permeability affected cell death induced by exogenous H_2_O_2_. Using cell viability analysis, we determined that increased membrane permeability had no effect on cell death while decreasing membrane permeability using cholesterol enhanced cell death significantly, as evidenced by decreased cell survival following transient exposure to H_2_O_2_ (Fig. 4). In summary, these data indicated that reactive oxygen species do not require entry into the cell in order to exert their cytotoxic effects.

As mentioned in the Mathematical Modeling section we ignore the peroxiredoxins to avoid complexity. This can be understood based on Eq. 4 and the fact that in our conclusions and results the major player was the proportionality factor between intercellular and extracellular H_2_O_2_ which only depends on permeability and catalase. This is due to the fact that catalase interacts directly with H_2_O_2_ without any intermediate steps. Since the chemical reactions involving the peroxiredoxins are similar to the GPxs, (in the sense that they detoxify H_2_O_2_ via intermediate steps) they do not appear in the proportionality term between intracellular and extracellular terms. Hence all of our results and conclusion remain valid.

The role of extracellular H_2_O_2_ has been investigated extensively. However, to the best of our knowledge based on a review of related literature, the cell killing behavior of extracellular H_2_O_2_ is associated with intracellular H_2_O_2_, not the effects from cell membrane^19^,^22^. This is due to the simple interpretation that an increase in extracellular H_2_O_2_ would increase the intracellular H_2_O_2_ and as a result invoke cell death. This assumption results in the neglect of the cell membrane’s role in this process. It may seem paradoxical that the cell membrane properties are involved in the cell killing (but not the intracellular H_2_O_2_) while the antioxidants located inside the cell could affect the cell killing. This paradox could be deciphered by looking at two channels which may be involved in reducing the effect of cell killing in the membrane. First, higher power of antioxidants lower the intracellular H_2_O_2_ and concurrently lower the concentration of H_2_O_2_ inside the cell membrane. Second, antioxidants such as glutathione could react with peroxidase lipids and reduce the damaging effect of these oxidants.

Understanding that the cell membrane may play a large role in cell killing opens new avenues for studying and understanding the diseases related to H_2_O_2_ such as cancer, diabetes, and Parkinson’s disease^23^,^24^,^25^. In many cases, the initiation of these diseases and their progression is associated with the generated intracellular H_2_O_2_ via the extracellular H_2_O_2_. This causes the role of the cell membrane to be neglected and hence many possibilities that could be considered for arresting and curing these diseases.

These findings also have a number of clinical implications. While it has been attractive to develop therapies that modify the levels of ROS as a mechanism of tumor cell death there has been a degree of controversy to effectively develop clinical strategies^26^. Our present work suggests that certain cell properties may potentiate greater cell death in tumor cell populations by modulation of ROS. Indeed, a number of membrane-selective transporters, such as aquaporins, have been known to facilitate the diffusion of ROS^27^. These cell surface proteins are often overexpressed on tumor cells^28^. Taking into considering our present findings, this might suggest that knowledge of aquaporin membrane permeability may affect response to ROS-inducing agents, such as ascorbic acid. Would overexpression of aquaporins create a greater extracellular concentration of ROS than low expressing cells and how might this affect a cells response to ROS-inducing agents such as ascorbic acid? Elucidating such questions may unravel why certain tumors and tumor cells are sensitive to ascorbic acid while others are not^29^.

Cell size is well-known to fluctuate over the course of the life of cells, coupling both the cell cycle and the metabolic state of mammalian cells^30^,^31^. It is also well-established that cell cycle and metabolic phenotype can determine various membrane permeability characteristics, such as the flux of freely permeant ions^32^ or the delivery of chemical agents with variable membrane permeation characteristics^33^. These evidences highlight the potential dynamism and variability that exists among the phenotypic landscape of cancer cells under therapy pressure, and adds greater complexity to our present approach. For example, could ROS-inducing agents, such as Ascorbic acid, induce greater toxicity when cells are in a particular cell cycle stage or metabolic state? Answers to questions such as these will drive a better understanding of drug treatment in cancer, building on the emerging paradigm of temporal and sequential sequence of drugs for cancer^34^.

In conclusion, we have employed a combination of theoretical modeling and experimental results to elucidate the mechanism behind the cancer cell killing ability of extracellular H_2_O_2_. This further clarifies the capability of pharmacological concentrations of ascorbic acid to selectively kill cancer cells. Our results demonstrate that there is no correlation between intracellular H_2_O_2_ and the cancer-killing trend in the range where killing by extracellular H_2_O_2_ is predominant. This provides a strong indication that cell membrane properties could provide the explanation for the cell killing properties of extracellular H_2_O_2_. This result has been validated by experiments that show cells treated with cholesterol exhibit lower intracellular H_2_O_2_ yet higher rates of cell killing. This study suggests a novel target for drug design that could reduce the risk of initiation and progression of various diseases.

## Additional Information

**How to cite this article**: Molavian, H. R. *et al*. Drug-induced reactive oxygen species (ROS) rely on cell membrane properties to exert anticancer effects. *Sci. Rep.* **6**, 27439; doi: 10.1038/srep27439 (2016).

## Supplementary Material

Supplementary Information

## References

[b1] Newman D. J. & Cragg G. M. Natural Products as Sources of New Drugs over the Last 25 Years. J Nat Prod. 70(3), 461–477 (2007). 17309302 10.1021/np068054v

[b2] Newman D. J. & Cragg G. M. Natural products as sources of new drugs over the 30 years from 1981 to 2010. J Nat Prod. 75(3), 311–335 (2012). 22316239 10.1021/np200906s PMC3721181

[b3] Chen Q. . Pharmacologic ascorbic acid concentrations selectively kill cancer cells: action as a pro-drug to deliver hydrogen peroxide to tissues. Proc Natl Acad Sci USA 102(38), 13604–13609 (2005). 16157892 10.1073/pnas.0506390102 PMC1224653

[b4] Chen Q. . Pharmacologic doses of ascorbate act as a prooxidant and decrease growth of aggressive tumor xenografts in mice. Proc Natl Acad Sci USA 105(32), 11105–11109 (2008). 18678913 10.1073/pnas.0804226105 PMC2516281

[b5] Cameron E. & Pauling L. Supplemental ascorbate in the supportive treatment of cancer: Prolongation of survival times in terminal human cancer. Proc Natl Acad Sci USA 73(10), 3685–3689 (1976). 1068480 10.1073/pnas.73.10.3685 PMC431183

[b6] Cameron E. & Pauling L. Supplemental ascorbate in the supportive treatment of cancer: reevaluation of prolongation of survival times in terminal human cancer. Proc Natl Acad Sci USA 75(9), 4538–4542 (1978). 279931 10.1073/pnas.75.9.4538 PMC336151

[b7] Moertel C. G. . High-dose vitamin C versus placebo in the treatment of patients with advanced cancer who have had no prior chemotherapy: a randomized double-blind comparison. N. Engl. J. Med. 312(3), 137–141 (1985). 3880867 10.1056/NEJM198501173120301

[b8] Padayatty S. J. . Intravenously administered vitamin C as cancer therapy: three cases. Can. Med. Assoc. J. 174(7), 937–942 (2006). 16567755 10.1503/cmaj.050346 PMC1405876

[b9] Hoffer L. . Phase I clinical trial of i.v. ascorbic acid in advanced malignancy. Ann. Oncol. 19(11), 1969–74 (2008). 18544557 10.1093/annonc/mdn377

[b10] Riordan H. D. . A pilot clinical study of continuous intravenous ascorbate in terminal cancer patients. P. R. Health Sci. J. 24(4), 269–76 (2009). 16570523

[b11] Chen Q. . Ascorbate in pharmacologic concentrations selectively generates ascorbate radical and hydrogen peroxide in extracellular fluid *in vivo*. Proc Natl Acad Sci USA 104(21), 8749–8754 (2007). 17502596 10.1073/pnas.0702854104 PMC1885574

[b12] Antunes F., Salvador A. & Pinto R. E. PHGPx and phospholipase A 2/GPx: Comparative importance on the reduction of hydroperoxides in rat liver mitochondria. Free Radic. Biol. Med. 19(5), 669–677 (1995). 8529927 10.1016/0891-5849(95)00040-5

[b13] Cairns R. A., Harris I. S. & Mak T. W. Regulation of cancer cell metabolism. Nat. Rev. Cancer. 11(2), 85–95 (2011). 21258394 10.1038/nrc2981

[b14] Gorrini C., Harris I. S. & Mak T. W. Modulation of oxidative stress as an anticancer strategy. Nat. Rev. Drug Discov. 12(12), 931–947 (2013). 24287781 10.1038/nrd4002

[b15] Raj L. . Selective killing of cancer cells by a small molecule targeting the stress response to ROS. Nature. 475(7355), 231–234 (2011). 21753854 10.1038/nature10167 PMC3316487

[b16] Klingelhoeffer C. . Natural resistance to ascorbic acid induced oxidative stress is mainly mediated by catalase activity in human cancer cells and catalase-silencing sensitizes to oxidative stress. BMC Complement. Altern. Med. 12(61), 1–10 (2012). 10.1186/1472-6882-12-61 PMC3404974 22551313

[b17] Chen P. . Anti-cancer effect of pharmacologic ascorbate and its interaction with supplementary parenteral glutathione in preclinical cancer models. Free Radic. Biol. Med. 51(3), 681–687 (2011). 21672627 10.1016/j.freeradbiomed.2011.05.031

[b18] Branco M. R. . Decrease of H_2_O_2_ plasma membrane permeability during adaptation to H_2_O_2_ in Saccharomyces cerevisiae. J. Biol. Chem. 279(8), 6501–6506 (2004). 14645222 10.1074/jbc.M311818200

[b19] Sousa-Lopes A. . Decreased cellular permeability to H_2_O_2_ protects Saccharomyces cerevisiae cells in stationary phase against oxidative stress. FEBS Lett. 578(1), 152–156 (2004). 15581633 10.1016/j.febslet.2004.10.090

[b20] Cooper R. A. Influence of increased membrane cholesterol on membrane fluidity and cell function in human red blood cells. J. Supramol. Struct. 8(4), 413–430 (1978). 723275 10.1002/jss.400080404

[b21] Nelson E. R., Chang C. & McDonnell D. P. Cholesterol and breast cancer pathophysiology. Trends Endocrinol. Metab. 25(12), 649–655 (2014). 25458418 10.1016/j.tem.2014.10.001 PMC4268141

[b22] Wang X., Simpkins J. W., Dykens J. A. & Cammarata P. R. Oxidative damage to human lens epithelial cells in culture: estrogen protection of mitochondrial potential, ATP, and cell viability. Invest Ophthalmol Vis Sci. 44(5), 2067–2075 (2003). 12714645 10.1167/iovs.02-0841

[b23] Whittemore E. R., Loo D. T., Watt J. A. & Cotmans C. W. A detailed analysis of hydrogen peroxide-induced cell death in primary neuronal culture. Neuroscience. 67(4), 921–932 (1995). 7675214 10.1016/0306-4522(95)00108-u

[b24] Evans J. L., Maddux B. A. & Goldfine I. D. The molecular basis for oxidative stress-induced insulin resistance. Antioxid Redox Signal. 7(7–8), 1040–1052 (2005). 15998259 10.1089/ars.2005.7.1040

[b25] Trachootham D., Alexandre J. & Huang P. Targeting cancer cells by ROS-mediated mechanisms: a radical therapeutic approach. Nat Rev Drug Discov. 8(7), 579–591 (2009). 19478820 10.1038/nrd2803

[b26] Wang J. & Yi J. Cancer cell killing via ROS: to increase or decrease, that is the question. Cancer Biol. Ther. 7(12), 1875–1884 (2008). 18981733 10.4161/cbt.7.12.7067

[b27] Bienert G. P. . Specific aquaporins facilitate the diffusion of hydrogen peroxide across membranes. J. Biol. Chem. 282(2), 1183–1192 (2007). 17105724 10.1074/jbc.M603761200

[b28] Ribatti D. . Aquaporins in cancer. Biochim. Biophys. Acta 1840(5), p. 1550–1553 (2014). 24064112 10.1016/j.bbagen.2013.09.025

[b29] Ohno S. . High-dose vitamin C (ascorbic acid) therapy in the treatment of patients with advanced cancer. Anticancer Res. 29(3), 809–815 (2009). 19414313

[b30] Dolfi S. C. . The metabolic demands of cancer cells are coupled to their size and protein synthesis rates. Cancer & Metabolism 1, 20 (2013). 24279929 10.1186/2049-3002-1-20 PMC4178206

[b31] Lloyd D. R., Holmes P., Jackson L. P., Emery A. N. & Al-Rubeai M. Relationship between cell size, cell cycle and specific recombinant protein productivity. Cytotechnology 34, 59–70 (2000). 19003381 10.1023/A:1008103730027 PMC3449736

[b32] Bubien J. K., Kirk K. L., Rado T. A. & Frizzell R. A. Cell cycle dependence of chloride permeability in normal and cystic fibrosis lymphocytes. Science 248, 1416–1419 (1990). 2162561 10.1126/science.2162561

[b33] Candipan R. C. & Sjostrand F. S. Permeability changes in isolated liver mitochondria during different metabolic states. Journal of Ultrastructure Research 89, 212–222 (1984). 6544887 10.1016/s0022-5320(84)80016-7

[b34] Goldman A. . Temporally sequenced anticancer drugs overcome adaptive resistance by targeting a vulnerable chemotherapy-induced phenotypic transition. Nature Communications 6, 6139 (2015). 10.1038/ncomms7139 PMC4339891 25669750

