# Supplementary material for: Drug-induced reactive oxygen species (ROS) rely on cell membrane properties to exert anticancer effects
Source: Sci Rep. 2016 Jun 9;6:27439. doi: 10.1038/srep27439 (PMC4899687; doi:10.1038/srep27439)
Supplement: Supplementary Information [file srep27439-s1.pdf]

Drug-induced reactive oxygen species (ROS) rely on cell membrane properties to exert anticancer effects.

Hamid R. Molavian[1,*,#], Aaron Goldman[2,3,4,#], Colin J. Phipps[1], Mohammad Kohandel[1,*], Bradly G. Wouters[5,6] , Shiladitya Sengupta[2,3,4] and Sivabal Sivaloganathan[1,7]

Mathematical Model

We use the chemical reactions for detoxifying H_2O_2 by catalase and glutathione peroxidase to derive the steady state concentration of H_2O_2 as a function of the other involved species.

Here we denote the intracellular concentrations of GSH, GPX1, GPX0, [GS-GPX], H_2O_2 and catalase by C_{gsh}, C_{gpx1}, C_{gpx0}, C_{gsgpx}, C_{h2o2} and C_{cat} respectively, and the total intracellular production rate of H_2O_2 by P_{h2o2}. Assuming constant concentrations of catalase and GSH are maintained, the kinetic equations, based on the aforementioned chemical reactions, for H_2O_2, GPX1, GPX0, and [GS-GPX] read,

$$\frac{dC_{h2o2}}{dt} = -k_1 C_{gpx1} C_{h2o2} - k_{cat} C_{cat} C_{h2o2} + P_{h2o2} \tag{1A}$$

$$\frac{dC_{gpx1}}{dt} = -k_1 C_{gpx1} C_{h2o2} + k_3 C_{gsh} C_{gsgpx} \tag{2A}$$

$$\frac{dC_{gpx0}}{dt} = k_1 C_{gpx1} C_{h2o2} - k_2 C_{gsh} C_{gpx0} \tag{3A}$$

$$\frac{dC_{gsgpx}}{dt} = -k_3 C_{gsgpx} C_{gsh} + k_2 C_{gsh} C_{gpx0}. \tag{4A}$$

Here and in the remainder of the paper we use the same notation as Ref. [15] and thus we automatically consider any synergistic effects between catalase and glutathione peroxidase.

The total production rate of H_2O_2 inside the cell, P_{h2o2}, can be written as follows (Eq. (2)),

$$P_{h2o2} = P^{int}_{h2o2} + P^{ext}_{h2o2} = P^{int}_{h2o2} + \mu \frac{A}{V} (C^{ext}_{h2o2} - C_{h2o2}), \tag{5A}$$

We are interested in finding the steady state solutions of Eqs. (1A) to (4A) where the production rate is given by Eq. (5A). We set the time derivatives equal to zero and solve equations (1A) to (4A), to obtain the following equation,

$$C_{h2o2} = \frac{P_{h2o2}}{k_1 C_{gpx1} + k_{cat} C_{cat}} = \frac{P^{int}_{h2o2} + \mu \frac{A}{V} (C^{ext}_{h2o2} - C_{h2o2})}{k_1 \left(C^0_{gpx} - \frac{P^{int}_{h2o2} + \mu \frac{A}{V} (C^{ext}_{h2o2} - C_{h2o2}) - k_{cat} C_{cat} C_{h2o2}}{k_2 C_{gsh}} \right) + k_{cat} C_{cat}} \tag{6A}$$

This equation can be rearranged to give the following quadratic equation for H_2O_2 in terms of the other parameters involved,

$$\alpha (C_{h2o2})^2 + \beta C_{h2o2} - \gamma = 0 \tag{7A}$$

$$\begin{cases} \alpha = k_1(k_{cat}C_{cat} + \frac{A}{V}\mu) \\ \beta = k_1k_2C_{gpx}^0C_{gs\hbar} + k_2(k_{cat}C_{cat}+\mu)C_{gs\hbar} - k_1(P_{\hbar 2o2}^{int} - \frac{A}{V}\mu C_{\hbar 2o2}^{ext}) \\ \gamma = k_2(P_{\hbar 2o2}^{int} - \frac{A}{V}\mu C_{\hbar 2o2}^{ext})C_{gs\hbar} \end{cases}$$

We can analytically solve this quadratic equation to obtain the following expression for the concentration of H_2O_2,

$$C_{\hbar 2o2} = \frac{|k_1k_2C_{gpx}^0C_{gs\hbar}+k_2(k_{cat}C_{cat}+\mu)C_{gs\hbar}-k_1(P_{\hbar 2o2}^{int}-\frac{A}{V}\mu C_{\hbar 2o2}^{ext})|}{2k_1(k_{cat}C_{cat}+\mu)}\left(\sqrt{1+\frac{4\alpha\gamma}{\beta^2}} - sgn(\beta)\right) \quad (8A)$$

In the limit of high concentrations of H_2O_2, which is of particular interest to us, Eq. (8A) can be reduced to

$$C_{\hbar 2o2} = \frac{k_1\frac{A}{V}\mu C_{\hbar 2o2}^{ext}+k_1P_{\hbar 2o2}^{int}-k_2(k_{cat}C_{cat}+\frac{A}{V}\mu)C_{gs\hbar}-k_1k_2C_{gpx}^0C_{gs\hbar}}{k_1(k_{cat}C_{cat}+\frac{A}{V}\mu)}. \quad (9A)$$

Supplemental Figure

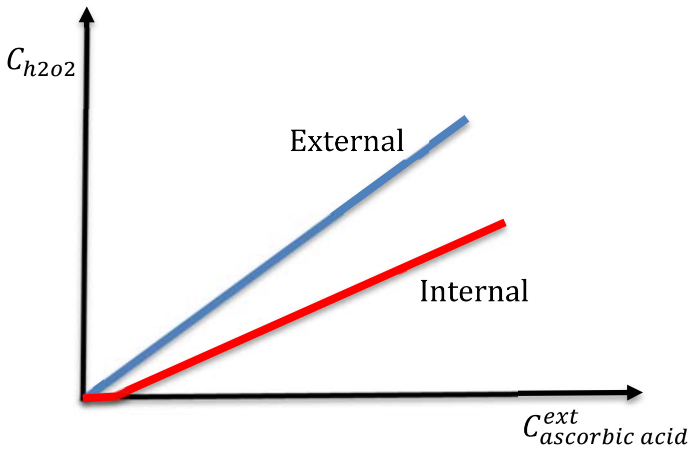

Fig. 1S: Concentration of external extracellular (blue) H2O2 and intracellular H2O2 as a function of the concentration of ascorbic acid.

Supplemental Table

Table 1S: List of reaction rates.

k_{cat}	k_1	k_2	k_3
$1.0 \times 10^7 M^{-1}s^{-1}$	$2.1 \times 10^7 M^{-1}s^{-1}$	$4 \times 10^4 M^{-1}s^{-1}$	$1 \times 10^7 M^{-1}s^{-1}$
